# A validated agent-based model to study the spatial and temporal heterogeneities of malaria incidence in the rainforest environment

**DOI:** 10.1186/s12936-015-1030-7

**Published:** 2015-12-22

**Authors:** Francesco Pizzitutti, William Pan, Alisson Barbieri, J Jaime Miranda, Beth Feingold, Gilvan R. Guedes, Javiera Alarcon-Valenzuela, Carlos F. Mena

**Affiliations:** Universidad San Francisco de Quito, Diego de Robles, s/n, Cumbayá, Ecuador; Duke University, 310 Trent Drive, Room 227, Box 90519, Durham, NC 27708 USA; Instituto de Geociências-IGC Belo Horizonte, Universidade Federal de Minas Gerais, Belo Horozonte, Brazil; Oswaldo Cruz Foundation (FIOCRUZ), Universidad Peruana Cayetano Heredia, Lima, Peru; Department of Environmental Health Sciences, School of Public Health, University at Albany, State University of New York, 1 University Place GEC, 145 Rensselaer, New York, NY 12144 USA; College of Economics Departamento de Demografia/FACE/UFMG, Office 3093, Av. Antônio Carlos, 6627-Pampulha, Belo Horizonte, Minas Gerais 31270-901 Brazil

**Keywords:** Agent-based model, Malaria, Amazon, Low endemicity, *Anopheles darlingi*, *Plasmodium vivax*, *Plasmodium falciparum*

## Abstract

**Background:**

The Amazon environment has been exposed in the last decades to radical changes that have been accompanied by a remarkable rise of both *Plasmodium falciparum* and *Plasmodium vivax* malaria. The malaria transmission process is highly influenced by factors such as spatial and temporal heterogeneities of the environment and individual-based characteristics of mosquitoes and humans populations. All these determinant factors can be simulated effectively trough agent-based models.

**Methods:**

This paper presents a validated agent-based model of local-scale malaria transmission. The model reproduces the environment of a typical riverine village in the northern Peruvian Amazon, where the malaria transmission is highly seasonal and apparently associated with flooding of large areas caused by the neighbouring river. Agents representing humans, mosquitoes and the two species of *Plasmodium* (*P.falciparum* and *P. vivax*) are simulated in a spatially explicit representation of the environment around the village. The model environment includes: climate, people houses positions and elevation. A representation of changes in the mosquito breeding areas extension caused by the river flooding is also included in the simulation environment.

**Results:**

A calibration process was carried out to reproduce the variations of the malaria monthly incidence over a period of 3 years. The calibrated model is also able to reproduce the spatial heterogeneities of local scale malaria transmission. A “what if” eradication strategy scenario is proposed: if the mosquito breeding sites are eliminated through mosquito larva habitat management in a buffer area extended at least 200 m around the village, the malaria transmission is eradicated from the village.

**Conclusions:**

The use of agent-based models can reproduce effectively the spatiotemporal variations of the malaria transmission in a low endemicity environment dominated by river floodings like in the Amazon.

## Background

The Amazon behaves as a complex system [1, 2], where socioeconomic, ecological and biophysical interactions occur between the human and the natural environments. In the last decades, part of the Amazon environment has been exposed to radical changes and severe alterations of native ecosystems ecologic equilibrium were generated [3]. In the northern Peruvian Amazon, the driving force of this change has been the influx of new immigrants that caused the rural frontier expansion [4]. In this context, activities such as the swidden-fallow agriculture have transformed the primary forest cover into a mix of cleared areas and secondary forest regrowth spots [5, 6]. This land cover modification process produced the variation of species composition of mosquito malaria vectors. The extension of riverine or floodable areas only partially covered by secondary regrowth generated new breeding areas for a highly competent malaria vectors, such as *Anopheles darlingi* [7, 8]. The reappearance of *A. darlingi*, that had been considered eradicated in the decades before the 1990, was accompanied, during the period from 1990 to 2000, by a remarkable rise of both *Plasmodium falciparum* and *Plasmodiumvivax* malaria in the Northern Peruvian Amazon [9].

Malaria is a vector-borne parasitic disease caused by the *Plasmodium* protozoa that are transmitted to humans through the bites of infectious mosquitoes of the genus *Anopheles*. Malaria is widespread around the world with nearly one half of the world population at risk, an estimate of 198 million cases and 584,000 deaths in 2013 [10]. Many elements interacting at several spatial and time scales contribute to the malaria infection dynamics. Environmental factors, such as climate, presence and characteristics of water bodies, weather, economic activities and social conditions, interact with individual-based characteristics and behaviours of mosquitoes and humans to give rise to complex spatial and temporal patterns. Barbieri et al. [11] describe how the complex interactions between economic land uses (*garimpo*, or small-scale gold mining, urban activities and agricultural activities) and population characteristics may determine distinct risk profiles of malaria prevalence in the Brazilian Amazon. Furthermore, every individual of the involved populations has its own life trajectory, which is partly determined by environmental factors, and that in turn alters the environment and also contributes to determine the life trajectory of other individuals.

The complex network of interactions involved in malaria transmission, generates feedbacks, non-linear behaviours and heterogeneities that are not easy to handle within ordinary mathematical models based on differential equations. Standard mathematical models of malaria transmission that evolved from the original formulation of Ross and MacDonald [12, 13] are based on a compartmental structure [14]. In such models, individuals are categorized in homogeneous groups and differential equations control the transitions from a compartment to another one. Although some compartmental model have tried to abstractly handle spatial heterogeneities [15], for the sake of simplicity compartmental models are usually based on the assumption of homogeneity inside the same compartment. Crucial factors in determining the malaria transmission, such as relative positions of houses and water bodies, environmental heterogeneities or individual-based behaviours are really difficult to be represented in an epidemiological compartment model. The natural approach to handle individual-based properties and spatial heterogeneities characteristic of the vector borne infection transmission is to make use of spatially explicit agent-based models (ABM). In ABMs the individuals are represented explicitly in a simulation code as agents interacting among them and with a representation of the natural environment [16]. In the malaria transmission study field, spatially explicit ABMs have been used to simulate the effect of management scenarios of aquatic mosquitoes habitats [17], to study the coupling of the hydrologic dynamics with the mosquito density [18], to study the spatial heterogeneities of mosquitoes distribution around water pools in a Shaelian village [19], to assess the risk of malaria re-emergence in the southern France [20] and to perform other similar analysis [21, 22].

The ABM presented in this paper integrates geographical analysis with entomology, demography and epidemiology in a simulation tool, able to reproduce the local scale malaria incidence dynamics in a small riverine village of nearly 1400 inhabitants located in the northern Peruvian Amazon. This study is a first attempt to represent through a validated mechanism-based simulation the local scale malaria infection dynamics mediated by *A. darlingi* in a region dominated by the flooding of a river as typical in the Amazon and in other tropical regions. The ABM is data-driven and validated: environmental data freely available from Internet are combined with field epidemiological observations to design a simulation tool able to reproduce the spreading of malaria that is disseminated by mosquito throughout the human population.

### Purpose of the model

The first objective of the presented ABM is to represent the relationships, feedbacks, and behaviours involved in malaria transmission, observed in a typical Amazon environment. This objective consists in demonstrating that the mechanisms and factors that drive the dynamics of the malaria epidemics in the study area, during the study period, are included in the model in such a way that the main emergent patterns of the real world system are reproduced. One of the primary mechanism-based explanations of the present model consists in demonstrating that the observed temporal changes in the malaria incidence can be reproduced through a correct representation of the flooding generated by the river level changes. Accordingly, one the most important components of the model are the calibration and the validation processes through which the simulation outputs are validated against real world epidemiological data. The second objective of the model is to study the dependence of the malaria risk on spatial heterogeneities, which are linked to the heterogeneous distribution of water bodies around the houses where people live. A third objective of the model is to study the effect of possible management strategies of the aquatic mosquitoes breeding sites around the village. An additional objective of this ABM is to build a baseline for further data collection and model developments which in the future will better account for complexity of the relationship between environmental, entomological and epidemiological factors in determining the malaria transmission dynamics at a local scale.

## Methods

### Short model description

**Fig. 1:**
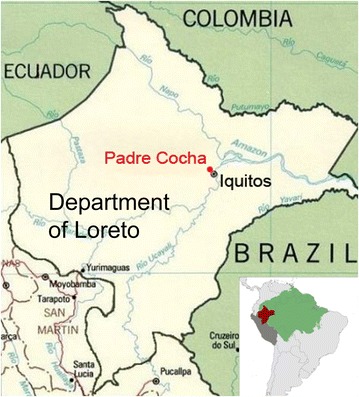
Location of the village of Padre Cocha near Iquitos. Iquitos is the capital of the Loreto department in the Northern Peruvian Amazon. In the *small inset* the Department of Loreto location (*red*) is showed respect to the rest of Peru (*dark grey*) and to the rest of the Amazon (*green*)

**Fig. 2:**
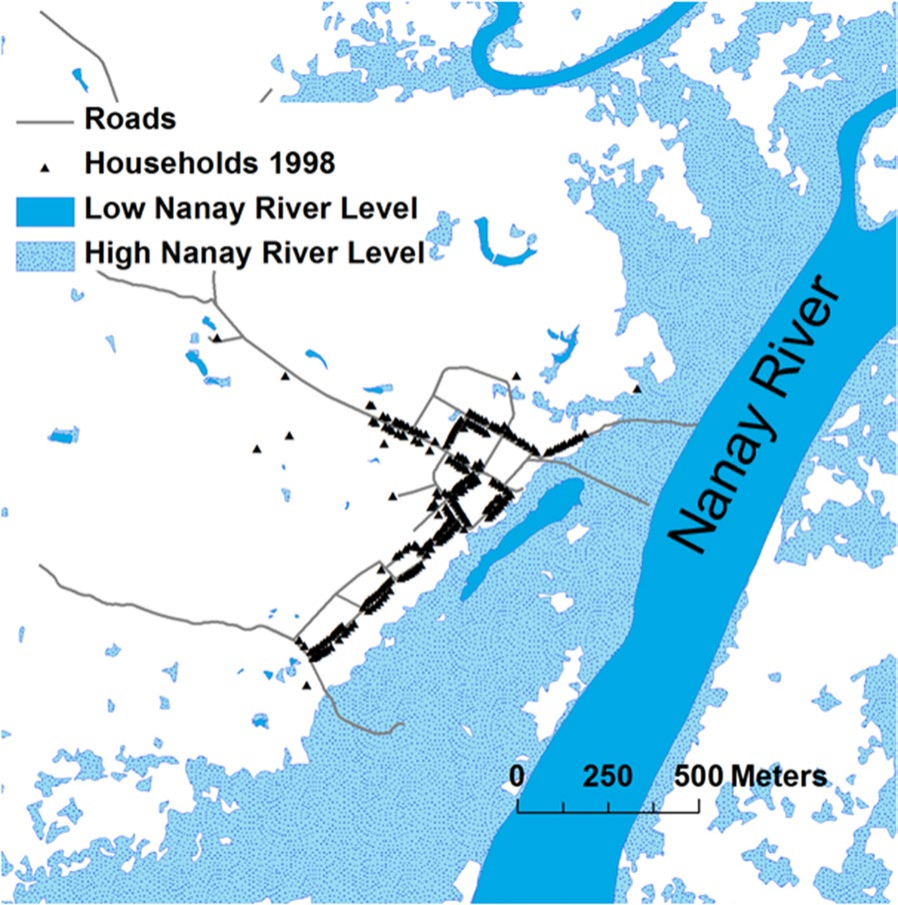
The simulation area. Water covers corresponding to low and high Nanay river levels around Padre Cocha village are represented in *different colors*. The Nanay River low level water cover corresponds to the digitalization of a satellite image taken the 12 of September 2013 while the high level water cover correspond to an image taken the 24 of March 2013. The household georeferenced positions of the year 1998 are from the paper of Bautista et al. [27]. The entire area showed in the figure is included in the simulation area

The ABM presented here is designed to reproduce the malaria transmission dynamics in a small, typical Amazon riverine village named Padre Cocha located in the Department of Loreto in the northern Peruvian Amazon (see Fig. 1). As it is showed in Fig. 2, the Nanay River, which flows nearly 500 m far from the village, as usual in the Amazon shows remarkable seasonal level variations (an average of 10 m yearly change). These changes produce floods that seasonally cover nearly half of the territory around Padre Cocha. The malaria transmission in Padre Cocha is strongly influenced by the seasonal river floodings because the flooded areas are ideal aquatic habitats for mosquitoes. The variations in the flooded area’s extension produce changes in the mosquito density and consequently in the malaria incidence. The model includes a number of elements that are considered important [17, 18, 21, 23] in determining the malaria transmission characteristics such as: environment features, biology, ecology and ethology of the mosquito vector, human host behaviour, asymptomaticity and Plasmodium response to anti-malarial treatment. Mosquito and human agents interact among themselves and with a spatial explicit representation of the area around the study village. The agents representation includes features describing the malaria infection state and the relevant aspects of the life history of every individual mosquito and human. The environment representation includes climate (rain and temperature), changes of the water cover, the distribution of households in the village and a digital elevation model. Due to the nocturnal biting behaviour of *A. darlingi*, humans are represented as agents located inside their respective houses. When an infectious mosquito bites a susceptible human, the human becomes infected and starts to progress through the different malaria infection stages, passing from a prepatent stage to the infectious, the symptomatic and finally to the recovered stage. Furthermore, if the mosquito is susceptible and the human is infectious, the mosquito starts to develop the *Plasmodium* infection. A mosquito can become infectious if it lives long enough to complete the *Plasmodium* maturation inside its body (sporogonic cycle). A mosquito never recovers from the infection. The mosquitoes are designed to reproduce the characteristics of the *A. darlingi* that is the main malaria vector in the study area. The two species of malaria *Plasmodium*, *P. falciparum* and *P. vivax*, observed in the study area during the study period are included in the model.

### Process overview and scheduling

The time step of the model is 1 h. Every 12 h, the mosquito agents emerge from the simulation pixels covered by water where other mosquitoes laid eggs and where the water cover endured for a period of time longer than the mosquito aquatic development time. During their life in the simulation, the mosquito agents try to have blood meals and subsequently to lay eggs. To achieve these goals the mosquitoes, only during night time, move through the simulation area changing their position passing from one pixel of the mosquito grid cover to an adjacent one. To human agents are assigned fixed position corresponding to the house they belong. The *Plasmodium* agents are represented as living inside the body of human and mosquito agents, passing through several stages of development that correspond to different stages of the infection of both vectors and hosts. The water cover of the simulation area changes accordingly to the Nanay River level registered in the date corresponding to the simulation time: higher river levels inundate more extended areas.

### Model calibration and validation

The process of validation of a model could be defined as the process of verifying that the model produces outputs in line with the design objectives and also with empirical observations [24, 25]. Through the validation against empirical data it is possible to verify if the stylized representation of the real world implemented in the model is accurate enough to generate emergent behaviours that are similar to the real world system. The ABM outputs have been validated against the observed monthly series of malaria incidence in Padre Cocha during the period from the beginning of 1996 to the end of 1998. To validate the ABM a simple strategy was followed. The set of parameters in the model for which empirical data are not known were selected as elements of what is called the calibration vector [26]. Then a first reasonable calibration vector starting value was selected and 24 versions of the model assigning slightly different values to the calibration vector were built. Then simulated monthly malaria incidence resulting from the average over eight runs of every model version are compared with the observed historical data of monthly malaria incidence. The simulated yearly incidences separately for *P. vivax* and *P. falciparum* were also compared with the observed corresponding yearly incidences. The version of the model producing simulated outputs closer to the observed data was used as starting point to build 24 additional versions of the model. Then 24 more versions were produced starting from the model that gave up the best output and so on iteratively. To complete the calibration process 680 different values of the calibration vector were tested for a total number of 5440 independent simulation runs. The simulation outputs presented in the Results section are obtained as averages over 48 repeated runs of the calibrated model. This number of repetitions was chosen high enough to have statistical error on the main observables below the 5 %. Every production run starts with a 365 days equilibration period during which all the environmental parameters are constant and equal to the values of the first simulation day.

### Spatial analysis

Once the model was validated, the simulations outputs are compared with the observed spatial cluster of high malaria risk detected in the same study period between 1996 and 1998 by Bautista et al. in a retrospective surveillance study [27]. The spatial cluster analysis was carried out using the purely spatial statistic scan analysis developed by Kulldorff [28] and implemented in the program SaTScan [29]. The Kulldorff’s method uses circular windows of variable size. The circular windows are centered in the position of every house in the simulation area and the radius of the windows is changed continuously from 0 up to a maximum size to include the 10 % of the population at risk. The SaTScan program compares the malaria counts within the scan windows with the expected number of cases resulting from Monte Carlo simulations. Then the window that corresponds to the maximum likelihood is selected and the number of cases inside this window is compared with the null hypothesis of no significant clustering. The analysis is carried out supposing that the malaria counts in each household follow a Poisson distribution. When the null hypothesis is true, the expected number of cases is proportional to the number of people living in the house.

### Software and hardware environment

The model have been implemented in the MASON [30] environment. MASON is a free, Java-based, discrete-event, multi-agent simulation library core used to reduce the repetitive code writing effort necessary to develop an ABM. Every simulation run required approximately 5 CPU hours of one core of an Intel Core i7-800 processor.

### Environmental module

The environment in an ABM is the space where the agents live. The kind of interactions between the agents and the environment, are crucial in determining the type and the characteristics of the ABM emergent properties. The detailed list of the environmental module parameters are showed in Table 1.

**Table 1:** Environmental module parameters

Parameter name	Value	Notes and references
Simulation time step	1 h	
Study area extension	2370 × 2520 m	
Centroid of the study area bounding box	3°41′54.17″S, 73°16′43.24″W	
Geographical grid cover pixel size	10 m	Based on spatial imagery resolution
Mosquito grid cover pixel size	30 m	[32–35]
Geographical grid extension	237 × 252 pixels	
Mosquito grid extension	79 × 84 pixels	Periodic boundary condition applied
Number of mosquito agents generated per oviposition	6.6 mosquitoes agents/oviposition	[37]
Maximum number of mosquito agents generated per unit of breeding area every 12 simulation hours	0.16 mosquitoes agents/m^2^/12 h	Calibration parameter
Reduction factor of the number of mosquitoes generated per unit of breeding area per unit of time in permanent water covers	1.5	Calibration parameter

### Study area

The study area is limited by a rectangular bounding box of 2370 × 2520 m, centered over Padre Cocha [3°41′54.17″S, 73°16′43.24″W]. Padre Cocha is located 5.5 km from the city of Iquitos, the capital of the district Loreto in Peru. The land cover around Padre Cocha is typical of a peri-urban area in the Peruvian Amazon: much of the cover is constituted by cleared areas that mix with secondary forest growth. Part of the land around Padre Cocha is covered all throughout the year by permanent stretches of water that include a big lagoon (*cocha* in quecha language) situated in the south-east area of the village. Approximately 500 m from the village, flows the Nanay River, an affluent of the Amazon River. Mosquitoes find breeding sites in every permanent or seasonal stretch of water around the village including the cocha [31].

### Meteorological data

Padre Cocha shows a typical equatorial climate: the rainfall level is high all around the year and there is not a sharp distinction between dry and rainy season. The average annual rainfall is 2616 mm with highest monthly rainfall intensity in April (310 mm) and December (282 mm) and lowest rainfall intensity in August (165 mm). The average annual temperature is 26.7 °C with a limited variability range, as usual in the Amazon (21–33 °C). The average humidity is 90 % (59–100 %). The average wind speed is 1 m/s with peaks of 4 m/s. The Nanay river level grows from the month of November to the month of February, while it decreases from May to July. The period from March to May is the high level period of the Nanay River, whereas the period from August to October is the low level period. The meteorological data included in the implementation of the ABM are: the daily rainfall and the daily minimum and maximum temperature. All the daily meteorological data used in this paper were obtained from the records of the meteorological station number 843770 (SPQT) located close to the Iquitos airport, approximately 9500 m far from Padre Cocha.

### The simulation grid covers

The study area is covered by two distinct squared grids of different cell size. The first grid cover is called the mosquito grid cover, which is used to specify the mosquito agents positions. When mosquito agents are looking for blood meals or aquatic habitats, during night hours, they move every simulation time step from one mosquito grid pixel to another adjacent one. The mosquito grid cell size is calibrated to obtain flight ranges that are consistent with the majority of fields observation that report flight ranges for *A. darlingi* around 800 and 1500 m [32–35]. Periodic boundary conditions are imposed on the mosquito grid cover so that pairs of opposing sides of the bounding box area are topological equivalent. The use of periodic boundary conditions is equivalent to have periodic copies of the simulation village all around the central simulation area. These copies are separated in space by the size of the simulation domain (2370 × 2520 m) reproducing with good approximation the characteristics of the area around Padre Cocha where several peopled areas, including the extreme fringes of the Iquitos city, are scattered around the village at distances ranging from 2200 to 2600 m along all the directions. The remaining elements of the study area are projected over the second grid cover that is called the geographical grid cover. The spatial resolution of this grid is selected according to the spatial resolution of the GIS imagery employed to build the river flooding model subcomponent.

### Hydrological submodule

The hydrological submodule implements an algorithm that reproduces in a simple way the floodings generated by the seasonal rise and fall of the Nanay River level in the area around Padre Cocha. The algorithm of the hydrological module converts a Nanay river level into a corresponding water cover. The input data of the hydrological submodule are: the monthly series of the Nanay River levels during the study period [27], two high resolution satellite images of the study area and an elevation model of the study area [36]. The two high resolution satellite images were obtained from Google Earth and correspond respectively to dates where the level of the Nanay River was near its maximum and near its minimum.^1^ Through a GIS analysis of the two high resolution spatial images was extracted a digitalized representation of the water land cover in the period of the high and the low Nanay River level. The two covers are showed in Fig. 2.

The mechanism underlying the hydrological submodule is simple: every 24 h during the simulation, the recorded value of the Nanay River level is read from the corresponding time series. Then the pixels that are covered by water in the high Nanay River water cover image and that have an elevation below the recorded Nanay River level are marked as covered by water.

### Mosquitoes breeding sites

During the simulation, every geographical grid cover pixel covered by water is considered as suitable as breeding site with the exception of the central part of rivers where the water flow would flush out eggs and larvae. Mosquito agents are generated from aquatics breeding sites where other mosquito agents have previously laid eggs. The number of emergent mosquito per oviposition is determined by the model parameter: “Number of mosquito agents generated per oviposition”. This number depends on several factors like the mosquito species and the biophysical characteristics of the aquatic habitats. A recent study [37] conducted on *A. darlingi* laboratory colonies reported a ratio of 6.6 emergent adults per oviposition. Although the laboratory conditions could be really different respect to the natural environment this value is adopted for the simulations. To include in the model the aquatic habitats carrying capacity [23], a maximum number of emergent adults per unit of breeding site area per half day is set.

Several studies [38–40] reported that the mosquito density and the biting rate of *A. darlingi* during the periods corresponding to the low Nanay river level fall almost to zero. This occurrence may be due, among other things, to a minor suitability as aquatic habitats of permanent water covers than flooded areas. To reproduce this experimental fact in the model all the permanent water bodies generate a number of adult mosquito agents that is decreased respect to the number produced in the flooded areas by a factor whose value is specified by the state variable: “Reduction factor of the number of mosquitoes generated per unit of breeding area per unit of time in permanent water covers”.

### Entomological module

The *A. darlingi* shows high anthropophilic and endophagous behaviour [8]. In the Loreto region, *A. darlingi* has been observed to be the almost exclusive malaria diffusion vector [40], especially in rural areas [38] and when the natural environment is highly altered by human activities [41]. For this reason the entomological ABM module of this study has been designed and parameterized using what is actually known about the *A. darlingi* ecology, ethology and biology. Although other species of the genus *Anopheles* observed in Loreto are competent malaria vectors, the minor relative importance of these species during the study period in determining the process of malaria diffusion and the similarities with the *A. darlingi* species assure that representing all the mosquito agents in the ABM as *A. darlingi*, is a quite good representation of the individual-based malaria vector dynamics in the Amazon. The values of all the parameters presented in the entomological module are showed in Table 2.

**Table 2:** Entomological module parameters

Parameter name	Value	Notes and references
Aquatic stage development time	15 days	[51]
Random walk pixels weight host-seeking mode and blood-seeking mode	0.85	Calibration parameter
Biting hours	From 7 pm to 5 am	[38]
Probability to fail the bite	0–1	0 when the human agent is protected against malaria 1 when the human agent is not protected against malaria
Resting time after blood meal	4 h	[49]
Time required for eggs maturation after the blood meal	48 h	[51]
Daily survival probability function	$p_{s} = e^{{ - \frac{1}{{ - 4.4 + 1.3 \cdot T - 0.03 \cdot T^{2} }}}}$	[53]; T is the temperature
survival probability factor during a rainy day	0.7	Calibration parameter
Minimum rain of a rainy day	100 mm/day	Calibration parameter

### Mating

**Fig. 3:**
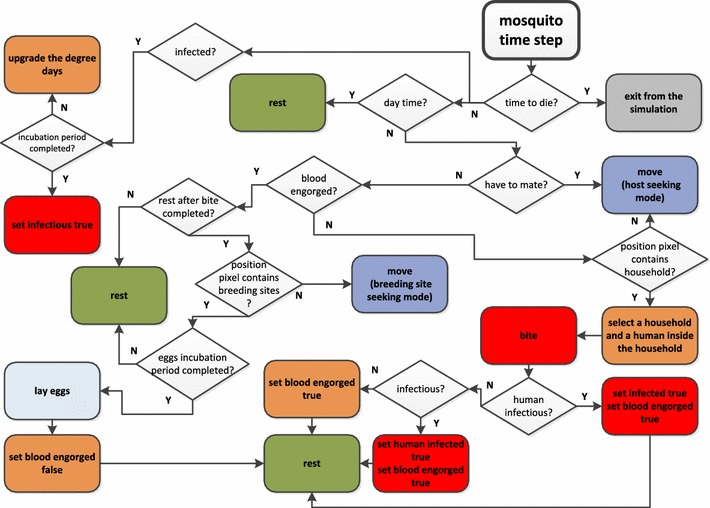
Flow chart of the mosquito agent time step

As noted, only the adult stage of mosquitoes is represented explicitly in the model and all the mosquito agents are females. In Fig. 3 the flow chart of mosquito agents life cycle as implemented in the model is showed. The first action that a mosquito agent accomplishes is mating. Mating behaviours among mosquitoes could be different from species to species [42]. Although little is known specifically about mating behaviour of *A. darlingi*, a study of releases and recaptures of *A. darlingi* mosquitoes conducted in Rôndonia, Brazil [43] showed that the mating of this species is not preceded by a swarm formation and occur possibly in the proximity of the blood seeking sites. Therefore a mosquito agent emerging from the breeding aquatic habitat is assigned to the blood seeking movement mode (see below).The probability distribution showed in Table 3 is used to decide when the agent will mate.

**Table 3:** Probability distribution of mating times after the emergence from the aquatic breeding site of mosquito agents, from: [43]

Time from the emergence from aquatic habitat (hours)	Probability of mating
12	0
24	0.1
36	0.5
48	0.3
60	0.1

### Blood-seeking and breeding habitat-seeking movements

After mating, the mosquito agent immediately starts to search a human agent to have a blood meal (blood-seeking movement mode). If the mosquito agent succeeds in having a blood meal, the agent enters in the breeding habitat-seeking movement mode to find an aquatic habitat to lay eggs. It is known [44] that the *Culicidae* family can shows long range responses to nonspecific olfactory substances like the carbon dioxide produced by animals respiration. Moreover mosquitoes showed to be responsive to long-range stimuli of olfactory host-specific substances. Different compounds from human body have been identified as specifically attractive for mosquitoes. Furthermore mosquitoes show vision of poor resolution but of high sensitivity also in night-time that enables them to visually differentiate open spaces from closed dark spaces like forests also from distances of 15–20 m. It is still unclear if mosquitoes can show cognitive abilities such as learning the location of the visited hosts, of resting and breeding site [45]. For this reason in this study all the mosquito agent memories about specific sites location are ignored. To represent the sensorial responses that guide the mosquitoes toward their human hosts and toward the breeding sites targets, the blood-seeking and the breeding habitat seeking movements are implemented in the simulation as weighted random walks [46]. In both movement modes the flight direction is completely random while the mosquito agent is far from the movement targets. On the other hand, when the mosquito agent is located in a grid cell adjacent to the targets, the movement is weighted to pull the mosquito agent toward the targets. A nearest neighbor grid cell of the mosquito agent location is weighted with a probability proportional to a weight *w* if it contains breeding sites or human households, and (*1* *−* *w*) in all the other cases (w є [0.5, 1]).

The influence of the wind over the mosquito host seeking and breeding site seeking movements is not included in the model. Despite the fact that for some mosquitoes species it was observed that a wind of 0.83 m/s may reduce the mosquito host-seeking flights [47], in a study of human-baited landing collection of *A. darlingi* mosquitoes conducted in Belize in a human altered rainforest environment [48], it was observed that even an average wind of 8.9 m/s do not alter significantly the number of female mosquitoes collected both outdoor and indoor during night hours.

### Blood meal

In the northern Peruvian Amazon, *A. darlingi* shows a nocturnal biting behaviour and two not pronounced biting peaks around dusk and dawn [38]. Therefore, a not blood-engorged mosquito agent that is located in a pixel populated by human agents waits until night time hours, from 7 pm to 5 am, to try to take a blood meal. If the mosquito is not located in pixels populated by human agents, it enters in the host-seeking mode and move until it finds a pixel populated by human hosts. Once the appropriate time for biting has come the mosquito agent randomly chooses one household inside its actual location pixel. Then the mosquito chooses at random a human agent associated to the selected household. The host-seeking process is repeated until the mosquito succeeds in having a blood meal. During the day-time hours, the mosquito agent rests and remains in the cell where it was actually located at 5 am. After the blood meal the mosquito agent always rests [49].

### Gonotrophic cycle and oviposition

Once the blood feeding process is complete, the ingested blood catalyzes the process of internal eggs development in the female mosquito [50]. The gonotrophic cycle is defined as the time elapsed from one blood-feeding to the next one and is composed by the time that is needed to the eggs maturation and the time from the oviposition to the next blood meal. The time required for eggs development could be considered constant in the temperature range of the Amazon and is about 48 h long [51]. The times from the oviposition to the blood-feeding and vice versa are not constant and depend on environmental variables because are determined also by the time taken by the host seeking and by the oviposition site seeking processes. After the bite, the mosquito agent switches to the breeding habitat-seeking movement mode to find an aquatic habitat to lay eggs. The agent moves until it finds a breeding site. If when the mosquito agent finds a breeding site the eggs maturation process is not completed, the agent waits in the same location until the oviposition.

### Aquatic development stage

The length of the mosquito aquatic development stage is in general a temperature dependent process. Nevertheless here this time is considered as constant within a temperatures range typical of the Amazon [52]. This development period is composed by the time needed to the eggs to hatching in larvae (48 h) plus the time needed for the development process from larvae to pupae and from pupae to the adult stage (13 days). As consequence, if a pixel of the geographical grid cover where a mosquito agent have laid eggs, maintains its water cover, at the end of the 15th day it will produce 6.6 adult mosquito agents per oviposition.

### Daily survival probability and death

The daily survival probability *p*_*s*_ of adult mosquitoes depends on many factors including the temperature. The classical relationship linking *p*_*s*_ with the temperature that is reported by Craig [53] is described by the following equation:

1 $$p_{s} = e^{{ - \frac{1}{{ - 4.4 + 1.3 \cdot T - 0.03 \cdot T^{2} }}}}$$

*T* is the daily average temperature. As observed by Monterio de Barros et al. [51] the *A. darlingi* daily survival probability can decrease during rainy days. For this reason in the model it is included a parameter called “survival probability factor during a rainy day” that sets the reduction of the survival probability during rainy days. A day is defined as a “rainy day” if the rainfall registered in this day is above a threshold specified by the model parameter “minimum rain of a rainy day”.

### Plasmodium module

Malaria is caused by a parasitic protozoan of the genus *Plasmodium*. The *Plasmodium* always has two hosts in its development cycle: a vector and a vertebrate host. In the case of *Plasmodium* species that cause malaria, the vector is a mosquito of the genus *Anopheles* and the vertebrate host could be a human as well as other mammal species like some monkeys. In the study area and in the Loreto Department, two different *Plasmodium* species are observed [9]: *P. falciparum* and *P. vivax*. Each species of *Plasmodium* give rise to different transmission pattern and different infection dynamics [54]. The values of the parameters presented in the entomological module are showed in Table 4.

**Table 4:** Plasmodium module parameters

Parameter name	*P. falciparum*	*P. vivax*	Notes and references
T_min_ extrinsic incubation	16 °C	14.5 °C	[55]; minimum temperature of incubation inside the vector body
DD extrinsic incubation	111 °C DD	105 °C DD	[55]; number of degree days to complete the sporozoites development inside the vector body
Intrinsic incubation time	(9–14) days	(12–17) days	[56, 57]; (min–max) range. The value is extracted from an uniform distribution between min and max
Transmission efficiency from asymptomatic human to mosquito	0.1	0.1	[65]
Transmission efficiency from human to mosquito	0.4	0.4	[64]
Transmission efficiency from mosquito to human	1	1	The value is chosen to maximize the number of infectious bites and reduce the simulations computational weight
Human infectious period if treated	300 h	24 h	[59]; calibration parameter only for *P. falciparum*
*P. vivax* recurrence time		203 days	[63]
*P. falciparum* recurrence risk		0.3	[63]
Gametocytemia time	(10–14) days	(9–13) days	[58–60]; (min–max) range. The value is extracted from an uniform distribution between min and max

### Mosquito-related *Plasmodium* parameters: the sporogonic cycle

The extrinsic incubation period is the only epidemiological feature in the model that originates from the interaction between mosquitoes and *Plasmodium* and is determined by the length of the sporogonic cycle. The sporogonic cycle or duration of the sporogony (DS) is the time required from the moment of the mosquito infection, to complete the sporozoite maturation in the salivary glands of the vector. This period depends on the *Plasmodium* species and also on the temperature:

2 $$DS = \frac{DD}{{T_{avg} - T_{min} }}$$

*T*_*avg*_ is the daily average temperature, *T*_*min*_ is the minimum temperature required for the development of the sporozoites and *DD* are the parasite specific number of degree days needed to the complete the DS. *T*_*min*_ and *DD* depend on the *Plasmodium* type [55].

### Human-related *Plasmodium* parameters

**Fig. 4:**
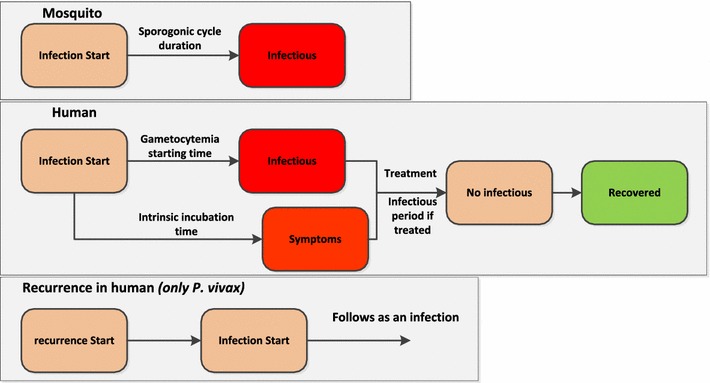
Development stages of the *Plasmodium* agent in the mosquito agent vector and in the human agent host

The human related *Plasmodium* parameters describe the progression of the malaria infection in human individuals. The human agent infection dynamics is more complex than the in case of the mosquito agent because human agents receive anti-malarial treatments and can recover from malaria. The *Plasmodium* agent development stages when interacting with human agents is showed in Fig. 4.

When a human is infected the *Plasmodium* agent starts its development process. The first development step, after the end of the intrinsic incubation period, is the transition from asymptomatic to symptomatic. In the real world this transition corresponds to the moment when the infected individuals go to the village health post to receive the treatment against malaria and the new malaria case is registered. Therefore, in the model when a human agent enters in the symptomatic stage the number of simulated malaria cases is upgraded. When the intrinsic incubation ends, the human agent is tagged as symptomatic. After the infection, when the “gametocytaemia time” is passed, the *Plasmodium* agent starts the gametocytaemia. Corresponding with the gametocytaemia onset the human agent became infectious and can transmit the infection to a mosquito agent. The parameter: “human infectious period” sets the length of parasitaemia after the beginning of treatment.

### Values of the human-related *Plasmodium* parameters

The World Health Organization reports that the intrinsic incubation period could be 7 days or longer [10]. The estimation for this parameter was based on previous publications which found incubation periods between 9 and 14 days for *P. falciparum* and 12–17 days for *P. vivax* [56, 57]. The infectious period for a human being corresponds to the period during which the infecting gametocytes, the sexual stage parasite, are present in peripheral blood and can access to a mosquito through a bite. Following the *Plasmodium* cycle, the appearance of gametocytes depends on the first wave of asexual parasites. The time necessary for gametocytaemia onset varies considerably among *Plasmodium* species. In the case of *P. falciparum* the releasing of mature gametocytes takes 8-10 days after which the gametocytes become infectious to mosquitoes in 2–3 days [58]. For that reason, in this model a value of 12 ± 2 days is used for the parameter “gametocytaemia starting time” in the case of *P. falciparum*. The production of *P. vivax* gametocytes starts with the first generation of merozoites and therefore gametocytaemia may occur within 1–3 days after the first asexual parasites are observed in the blood [59, 60], this is equivalent to the prepatent period plus 1–3 days that gives the 11 ± 2 days used in this model.

Considering treatment, *P. vivax* gametocytes are susceptible to most commonly used anti-malarial treatments with an approximate circulation time of 1 day after treatment and they disappear with asexual parasite clearance [59, 61]. This is reflected in the model parameter “human infectious period if treated” that is equal to 24 h for *P. vivax*. On the other hand, *P. falciparum* gametocytes have been found to circulate for up to 55 days after treatment unless primaquine is used, which reduces this time to approximately 6 days [62]. The average value used in this model for the *P. falciparum* parameter “human infectious period if treated” is 300 h which was based on the treatment regime used for Padre Cocha during the study period which included primaquine and sulfadoxine/pyrimethamine that eliminate parasitaemia in 7–14 days [31].

It must be considered that due to hypnozoite reservoirs in the liver, *P. vivax* infections present themselves in cycles and recurrent infections are common [56]. Both risk of recurrence and median survival time to recurrence were estimated to be of 0.3 and 203 days, respectively [63]. Even though the reported risk of recurrence tends to be higher in recent publications, at the time of the Padre Cocha study primaquine was being used and treatment resistance was low, therefore, risk of recurrence was lower than in current times.

### Transmission efficiency from humans to mosquitoes

Transmission efficiency of *Plasmodium spp.* from humans to *A. darlingi* mosquitoes has been determined in few studies, considering different factors such as asymptomaticity, parasite density and gametocytaemia. Bharti et al. [64] found a value around 35 % for transmission efficiency from human patients infected by *P. vivax* to *A. darlingi.* In the study presented by Alves et al. [65], although biased by the low number of observed transmissions, symptomless patients with very low parasite densities, approaching zero parasites per microlitre were found still infecting 1.2 % of the *A. darlingi* mosquito population. The same study found 22 % of symptomatic patients being able to successfully infect *A. darlingi* mosquitoes. In this model it is used a value of 0.4 for the transmission efficiency from symptomatic acute patients to mosquitoes for both *P. falciparum* and *P. vivax*. Given that asymptomatic patients are not as efficient transmitters and that their values for transmission efficiency are significantly lower, in this model the transmission efficiency from asymptomatic human agents to mosquitoes is set to 0.1 for both *P. falciparum* and *P. vivax*.

### Human module

Humans are represented in the model as individual agents. The *A. darlingi* mosquito has nocturnal biting habits therefore the representation of daily human activities is not included in the model and the human agents are located in their houses and do not move during the simulation. In the study period the human population of Padre Cocha have oscillated between 1396 and 1405 inhabitants [31]. The human agents are assigned randomly to the houses of the village. The number of human agent assigned to every household is determined by a Gaussian distribution. The minimum number of human agent in a house is set equal to 1. The positions of the houses in Padre Cocha during the study period are obtained from the study of Bautista et al. [27]. The values of the parameters of the human module are showed in Table 5.

**Table 5:** Human module parameters

Parameter name	Value	Notes and references
Average number of human agents	1400	[31]
Parameters of the Gaussian distribution of human agents in every house	Mean = 6 σ = 3	[31]
High incidence threshold	8 %	Calibration parameter
Low incidence threshold	1 %	Calibration parameter
Probability of adoption of protection methods	0.15	Calibration parameter
protection period duration	365 days	Calibration parameter
Pprotection period fraction	0.4	Calibration parameter
Fraction of human agents permanently protected against mosquito bites	0.3	Calibration parameter
Fraction of asymptomatic human agents	0.07	*P. falciparum*
Fraction of asymptomatic human agents	0.05	*P. vivax*

### Human behaviour

The human agent behaviour is designed to reproduce the changes in the adherence to methods of protection against mosquito bites. In what follows, any element that obstacles the mosquitoes in biting humans is considered as a protection method against mosquitoes bites (bed nets, repellants, protective clothing, burn coils, fumigants). Several studies in Africa and in the Amazon and in other malaria endemic areas [66, 67] demonstrated that behavioural aspects of human individual are important in determining the malaria infection dynamics. The adherence to the use of bed nets was found influenced in the Brazilian Amazon [68] by seasonal factors including the absence or the presence of mosquitoes. These studies seem to indicate that local populations adopt protection methods against mosquito bites basically only if the perception of the malaria risk or the annoyance caused by mosquito bites is high enough to compensate the discomfort and the economic costs associated with the use of bed nets and other protection methods. These facts are stylized in the ABM associating to the human agents a Boolean state variable called “protection against malaria” that specifies if the human agent is protecting itself from mosquito bites or not. When the malaria incidence in the village is high human agents tend to protect themselves from mosquito bites changing their status of protection against mosquito bites. When the malaria incidence is low the human agents will switch gradually to a no protection state. A portion of human agents never change the protection state and is always considered as protected.

**Fig. 5:**
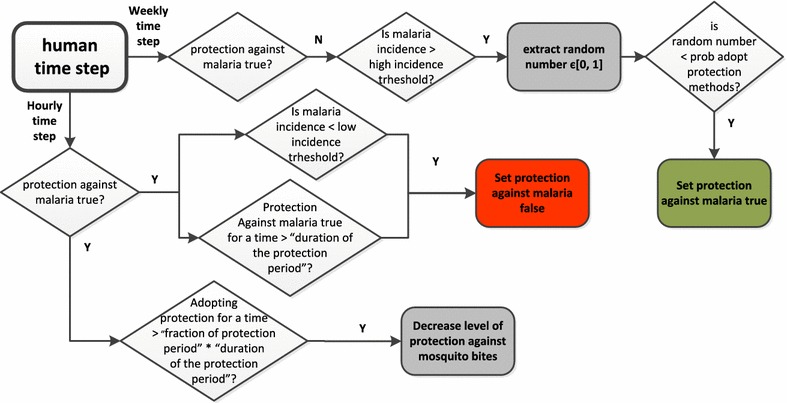
Flow chart of the human agent time step

The way human agents change their protection against mosquito bites state is represented as a flow chart in Fig. 5. If the malaria incidence is above the value specified by the variable “high incidence threshold”, every simulation week, the human agent changes the value of its “protection against malaria” state variable to true with a probability specified by the parameter “probability of adoption of protection methods”. The total adherence to the use of the protection methods against malaria is then maintained by the human agent for a period that is a fraction the time specified by the variable “protection period duration”. This initial fraction is given by the following relationship:

$$\begin{aligned} Period \,of \,total \,protection &= protection \,period \,duration \cdot \\ & \quad protection \,period \,fraction \end{aligned}$$

After the period of total protection the level of protection against mosquito bites is linearly decreased and the period of partial protection starts. This period of partial protection lasts until the end of the protection period. At the beginning of the partial protection period when *P*_*f*_ is equal to 0 every attempt of bite will fail. At the end of the protection period when *P*_*f*_ is 1 every bite attempt succeeds. The reduction of the value of the variable P_f_ is introduced in the model to represent the diminution of the adherence to protection methods as well as the reduction in time of the effectiveness of the prevention method itself (holes creation in bed nets, decrease in the content of insecticide in impregnated bed nets, etc.). The last possible action included in the human agents behaviour is the following: when the simulated malaria incidence falls below the value specified by the variable: “low incidence threshold” the human agent switches to a state of no protection against mosquito bites.

### Immunity and asymptomaticity

The model does not consider explicitly the dynamics of acquired immunity against malaria. Only a fixed portion of human agents is considered as immune, asymptomatic and constantly infectious. The study of Roper et al. in Padre Cocha during 1998 indicated that only 2 % of the asymptomatic population was positive for malaria [31]; nevertheless only microscopy techniques were used to determine this result. There is discrepancy between microscopy and PCR diagnosis, with poor performance of microscopy at low parasite densities [69]. In general, prevalence of asymptomatic infections in the Amazon region, including Peru, Brazil and Venezuela, is relatively low with values that are found to be less than 10 % [70] and around 5–8 % in Zungarococha and Manacamiri located in the peri-Iquitos area (Unpublished data presented in [70]). Since, as showed by Alves et al. [71], the PCR technique is 6–7 times more efficient than microscopy for detecting plasmodial infections the values of the fraction of asymptomatic human agents used in the model is six times higher of what found by Roper et al. All the asymptomatic human agents are considered as no transmission-blocking.

As mentioned by Roper et al. [31] no superinfection was detected in Padre Cocha during the study period. For this reason no superinfection is considered in the model: when a human agent is infected by a species of *Plasmodium*, the agent is considered as no susceptible to the infection of the other species of *Plasmodium* of the model.

## Results

### Cyclic peaks of malaria incidence

**Fig. 6:**
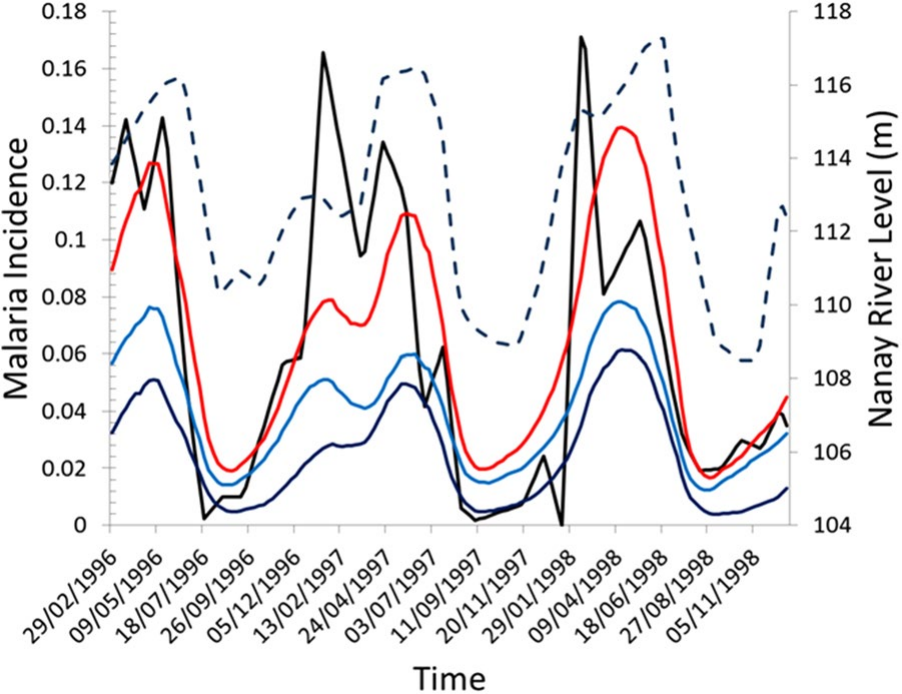
Monthly malaria incidence as calculated by the ABM (*red line*: total malaria incidence, *light blue* *P. vivax* malaria incidence, *dark blue* *P. falciparum* malaria incidence). The simulated malaria incidence curves are compared with the observed monthly malaria incidence (*black line*) and the Nanay river level (*blue dotted line*) in Padre Cocha, Loreto, Peru, during the period from the beginning of the year 1996 to the end of the year 1998. The observed incidence curve is obtained from the study of Bautista et al. [27]

The model output resulting from the calibration process is presented in this section. In Fig. 6 the curve of simulated monthly total malaria incidence is compared with the corresponding observed malaria monthly incidence. It is possible to note that the model can reproduce the observed pattern of temporal variation of the malaria incidence to a large extent. The sum of the *P. vivax* and *P. falciparum* incidences give rise to a simulated curve that, like the observed one, is almost zero during the months of August and September during the Nanay river low level season. The same simulated curve shows pronounced peaks corresponding to the Nanay river high level seasons from the month of March to the month of June.

Unfortunately, it is not possible to have access to the desegregated counts of the observed malaria monthly incidence for *P. vivax* and *P. falciparum* and the monthly incidence curves cannot be validated separately for *P. vivax* and *P. falciparum* malaria. The only available desegregated data [27] are the cumulative yearly malaria incidence separated for *P. vivax* and *P. falciparum* that are showed in Table 6 together with the simulated yearly incidences. Also in this case of the yearly malaria incidence the outputs of the model are close to the empirical observations.

**Table 6:** Observed and simulated yearly malaria incidence separated for *Plasmodium vivax* and *Plasmodium falciparum*

Year	Observed *P. falciparum* yearly incidence (95 % CI)	Simulated *P. falciparum* yearly incidence	Observed *P. vivax* yearly incidence	Simulated *P. vivax* yearly incidence
1996	0.16 (0.14–0.18)	0.28	0.51 (0.48–0.53)	0.50
1997	0.29 (0.27–0.32)	0.30	0.47 (0.44–0.49)	0.46
1998	0.12 (0.10–0.13)	0.35	0.56 (0.53–0.58)	0.53

The observed yearly incidence data are obtained from the study of Bautista et al. [27]

**Fig. 7:**
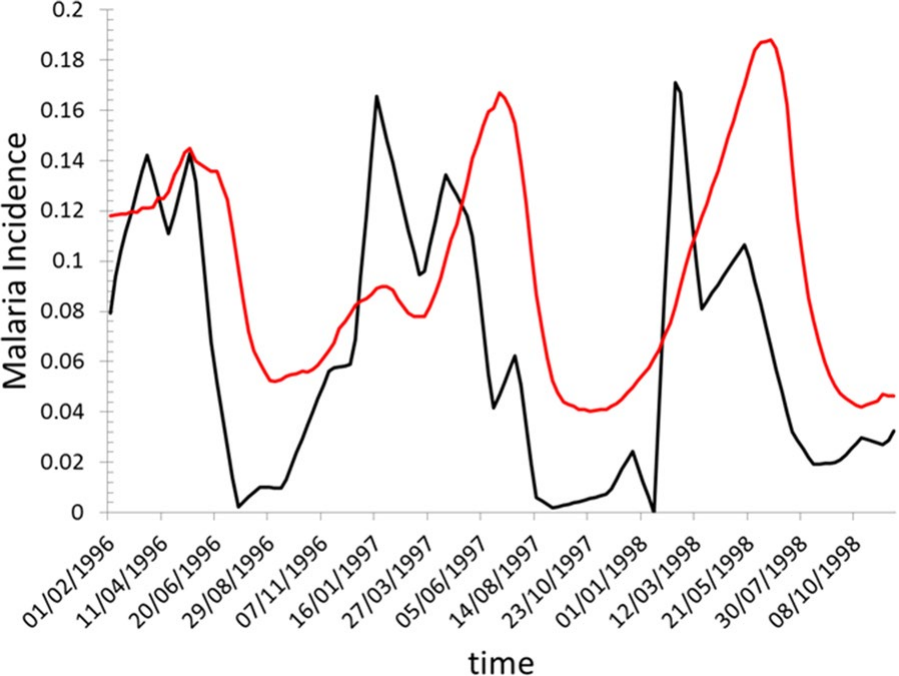
Monthly malaria incidence as calculated by the ABM in the case when the human agents do not change the adherence to protection method against mosquito bites. The simulated curve (*red*) is compared with the observed monthly malaria incidence (*black*) in Padre Cocha, Loreto, Peru, during the period from the beginning of the year 1996 to the end of the year 1998

To show how the model is sensible to the interactions between human individuals and the environment where they live, a modified version of the model is produced where the adherence level to methods of prevention against mosquito bites is maintained constant. In this new model parametrization the seasonal changes of the adherence to mosquito bites preventions methods are not included and the fraction of protected human agents is maintained equals to 0.46. As it is showed in Fig. 7, after the sharp increase in the malaria incidence that take place in August and September, the curves of simulated malaria incidence calculated without taking into account the human behaviour show a delay of nearly 2 months with regards to the empirical curve before the descendant phase. The same delay was not showed by the curves in Fig. 6 where the human agents behaviour is included. Also the simulated curves with no human behaviour included show pronounced minima in October and November but the absolute value of these minima is far from the value that the observed malaria curves take in the same months.

### Spatial analysis

**Fig. 8:**
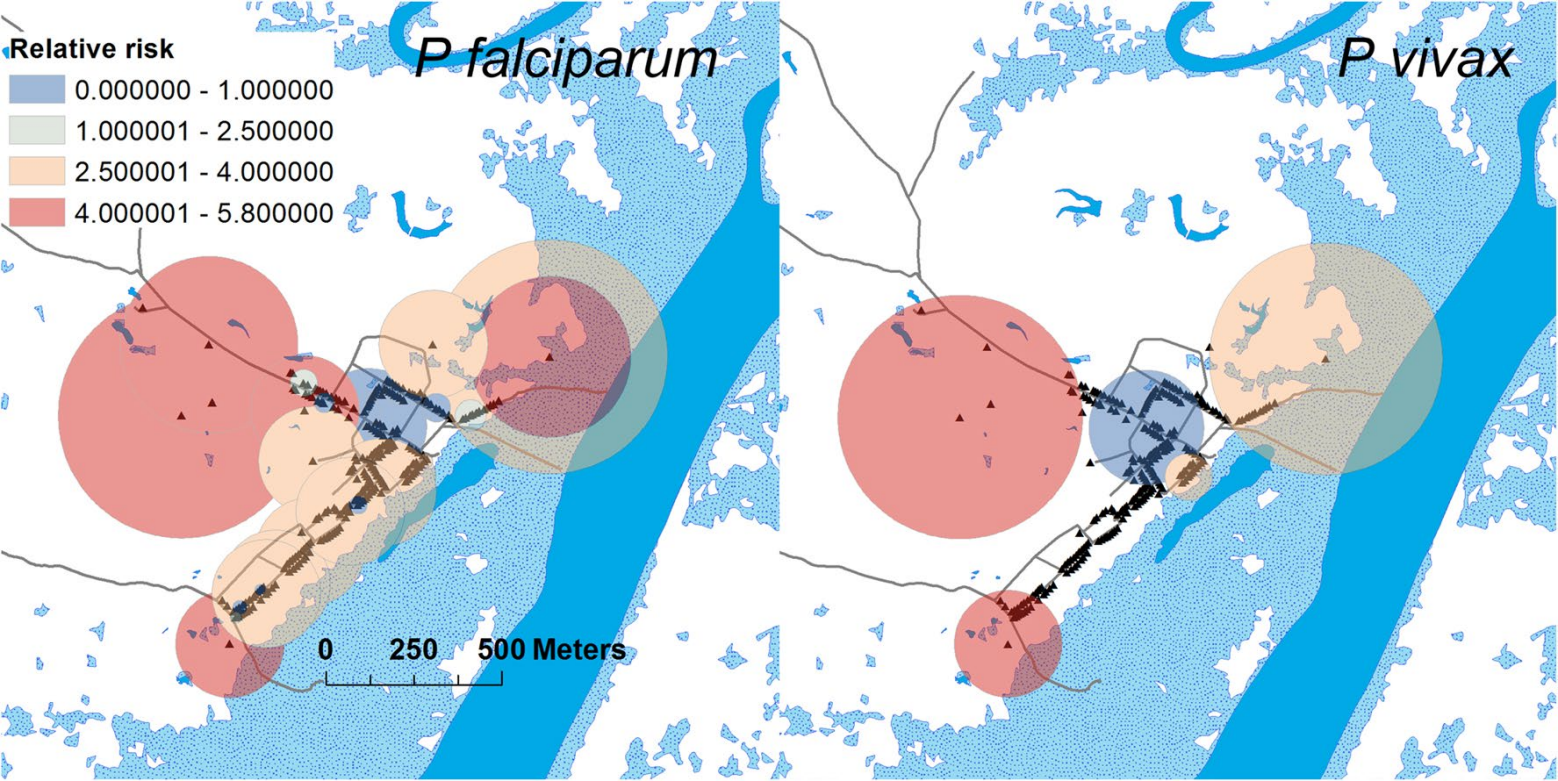
Clusters of relative risk of households in Padre Cocha. *Red circles* correspond to high relative risk; *blue circles* correspond to low relative risk

As can be seen in Fig. 8, the spatial cluster analysis evidenced significant clusters of both *P. vivax* and *P. falciparum.* The relative risk of these clusters is defined as the number of cases in a windows centered over a household observed during the simulation, divided by the number of expected cases in the same window. The range of variation of the relative risk is quite high, fluctuating from a maximum of 5.6 to a minimum of 0.01. For both *P. vivax* and *P. falciparum* clusters of high relative risk are observed for the households located closer to the permanent and seasonal mosquitoes breeding sites. In the central part of the village, far from water bodies there are clusters of low risk. The ABM simulation evidences also that isolated houses like those located on the northeastern and northwestern part of the village could be subjected to higher risk of malaria. In the case of *P. falciparum* it is possible to observe the presence of high relative risk clusters close to the floodable areas in the southeastern side of the village not observed for *P. vivax.* The significant spatial fluctuations caused by the households and larval habitats heterogeneous spatial distribution evidenced by the simulation are in good agreement with the results obtained from the same spatial analysis carried out on experimental data by Bautista et al. [27] whose study also evidenced the center and the borders of the village as areas of low and high relative risk, respectively.

**Fig. 9:**
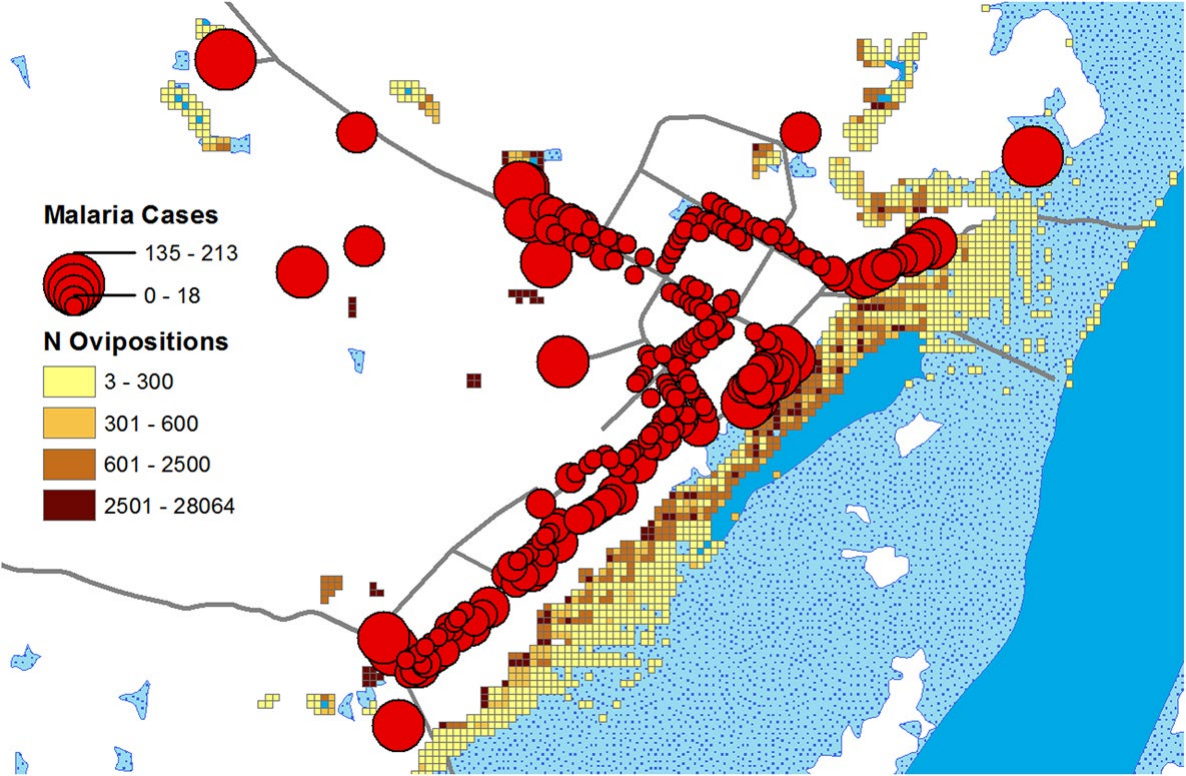
Oviposition sites and malaria cases during the simulation period. The households are showed as graduated *red circles* whose areas are proportional to the number of simulated malaria cases. The geographical grid cover pixels used as breeding site by the mosquito agents are showed in graduated *colors* to highlight the number of ovipositions

A closer view of relative positions of households, malaria cases and breeding sites is showed in Fig. 9. The breeding productive sites are located close to inhabited places predominantly along the inundated river bank close to the village southeastern side. A comparison between Figs. 2 and 9 shows that during the river low-level season only few mosquito agents laid eggs along the river bank and the most productive breeding sites during this season are the permanent stretches of water scattered around the village. The number of ovipositions in these permanent pools around the village is higher in proximity to the village. Furthermore, in agreement with the spatial analysis presented above, a great number of malaria cases among human agents are concentrated in households located close to the floodable areas in the southeastern part of the village and close to the permanent pools far from the river.

### Larval source management

The breeding sites management or larval source management (LSM) is an approach aimed at reducing the number of adult mosquito individuals emerging from breeding sites. The LSM can be implemented following various strategies, for example by manipulating the habitats eliminating standing water. A second strategy is the habitat alteration adding chemicals to water in order to kill or to prevent the development of the larvae [10]. Obviously when the land water cover changes remarkably as consequence of massive flooding originating from the changes of a river level like in the Amazon, adding chemical to flooded areas would be ineffective. In the other hand, many strategies of habitat manipulation are aimed to reduce the extension of aquatic habitats. As noted in the introduction to this paper, the malaria outbreaks observed in the Iquitos region from the year 1996 were correlated with anthropogenic activities that altered the mosquito habitats leading to a change in the mosquito species composition. Certain types of environment modifications amplify the presence of *A. darlingi*: areas associated with low level of shading resulting from deforestation, areas with large water bodies and areas close to human population [7, 41]. Moreover Turell et al. [38] found that in 1999 the density of *A. darlingi* in Puerto Almendra, a village 18 km far from Padre Cocha, located in a riverine environment very similar to Padre Cocha, resulted very high if measured inside the village, while the same density fell almost to zero if measured 300 m far from the village in a unaltered forested site. These facts may suggest that if the unaltered total cover of the original forested habitat is restored in critical areas around a village as a kind of LSM habitat manipulation, the resulting shading effect would decrease the presence of *A. darlingi* and the malaria transmission. Another habitat alteration strategy could be the construction of barriers to prevent the water of the river to inundate the areas close to the village. In any case the result of the LSM would be the elimination of mosquito breeding sites around the village.

The calibrated version of the ABM was used to simulate the effect of the elimination of mosquito breeding sites around the village of Padre Cocha. To carry out this test, several version of the model were built creating a sequence of “what if” scenarios. In these test simulations a buffer area is created around every house of the simulation. The buffer area is delimited by two radius r_b_ and R_b_. The first radius is fixed in every scenario simulation and is equal to 20 m. The second buffer radius is greater than r_b_ and changes in every test scenario. All the geographical grid cover pixels that are distant more than r_b_ and less than R_b_ from a house belong to the buffer area and the pixels belongings to the buffer area are considered as not suitable as breeding sites. Six independent simulations were then carried out varying the value of R_b_ from 50 to 300 m in order to observe the effects of larval habitats elimination on the malaria incidence.

**Fig. 10:**
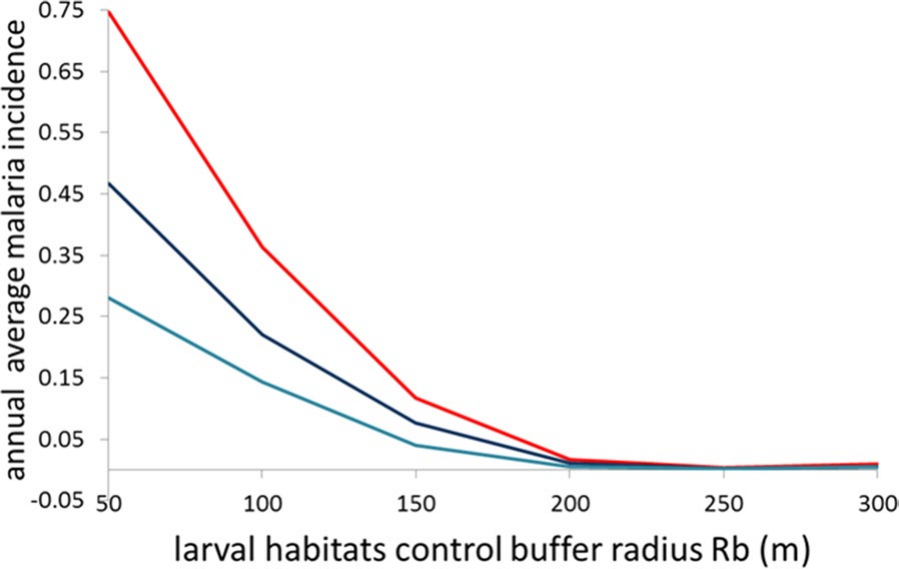
Simulated average yearly malaria incidence as function of the larval habitats control buffer radius R_b_. All the breeding sites inside the area delimited by the buffer radius are removed from the simulation. *Red line*: total yearly average malaria incidence, *dark blue line*: *P. vivax* yearly average malaria incidence, *light blue line*: *P. falciparum* yearly average malaria incidence

Figure 10 shows a plot resuming the results of the “what if” scenarios simulations. The total annual malaria incidence calculated as an average over the three (1996, 1997, 1998) simulation years considerably decreases with the increasing of R_b_. For R_b_ values above 200 m the malaria incidence fall almost to zero while for R_b_ = 150 m the incidence show a decreasing of the 87 %. This sharp effect is due to the fact that the mosquito agents have to increase the length of the blood-seeking and the breeding site-seeking times because with the increase of R_b_ the breeding sites are moving progressively further from the houses. This generates a gonotrophic cycle increase leading to a point where the average length of two gonotrophic cycles is greater than the average length of a mosquito agent life and the malaria transmission is blocked.

## Conclusions

The ABM presented in this paper reproduces the observed patterns of local scale malaria transmission dynamics in a small riverine village in the Northern Peruvian Amazon. As usual in agent-based modelling, a bottom-up strategy was followed: individual scale elements (human and mosquito agents) were assembled together with a macro scale environment description to create a model where macroscopic patterns emerge as a collective behaviour. On this regard the model offers an explicit representation of interactions between two populations of humans and mosquitoes in the context of a changing environment. The model was calibrated against empirical data of malaria incidence and the resulting calibrated model generated outputs that are in agreement with observed data. Obviously the model is not able to reproduce perfectly the malaria incidence time series and the observed spatial heterogeneities. Many factors not represented explicitly in the model contribute to the malaria transmission dynamics in the real world system. For example the exact relationships among the mosquito biology, ecology and ethology and environmental and climatic factors is far from being understood and studied in full detail. Consequently those interactions are represented only approximately in the model. For example the aquatic development stage of the mosquito life cycle is not represented in full details as shown in other individual-based models of malaria transmission [18, 23, 72]. A more detailed representation of *A. darlingi* subadult development stage would imply the use of an extended set of unknown parameters describing the aquatic habitat in terms of mosquito eggs, larvae, and pupae predation, death rates, biomasses, number of eggs per oviposition and habitats carrying capacity. In the presented model mosquitoes breeding sites are only characterized as areas where the mosquito agents lay eggs and from where adult mosquitoes emerge in number proportional to the ovipositions. Other aspects of mosquitoes life cycle are not included: mosquito agents are strictly anthropophilic and the possibility of blood meals from animals is not included. Also some aspects describing the malaria transmission process like the transmission efficiency from mosquito to human are no included in the model because the value determination for this parameter is really problematic. For that reason in the model, the malaria transmission efficiency from mosquitoes to symptomatic humans is considered equal to 1 although in the real world this efficiency could be significantly less than 1 [73]. Similarly the immunity acquisition and dynamics in humans are not represented in the model. With respect to the complex behaviours and features of humans as individuals and the socioeconomics features of the village under study, only a limited and approximate representation is included in the model. Patterns of human individual movements are not represented in the model although human work-related and regional human movements are identified as important driving forces in determining the malaria transmission process [74]. More specifically Roper et al. [31] showed that some of the daily human activities in Padre Cocha after 6:00 pm and before 6:00 am are associated with an increased malaria risk. The model calibration had possibly included implicitly these increased malaria risks tuning the overall degree of protection against mosquito bites of the human agent population.

The validated version of the model was used to build several “what if” scenarios to understand the effect of larval sources site management. This result can be considered as an example of how an ABM of local malaria transmission could be used as a tool to study malaria transmission and to build a malaria control planning system [75]. A number of scenarios were considered to calculate the malaria incidence as a function of the extension of the area around the study village where the mosquito aquatic habitats were eliminated. The simulated scenarios showed that, eliminating mosquito larva in a buffer area extended more than 200 m around the village the malaria transmission is completely eliminated. A similar “what if” scenario study was carried out by Gu and Novak [17] for *A. gambiae* in an ABM of a hypothetical village where human habitations and breeding sites were created at random over a squared 40 × 40 grid. In this work the elimination of aquatic habitats generated a slower malaria incidence decrease, respect to what presented here, giving a reduction of 94 % in malaria incidence when the breeding sites were eliminated in a range of 300 m from the houses. The same model gives a reduction of 86.8 % with a buffer radius of 200 m. Although the flight ranges of mosquitos agent are similar in the model presented here respect to the model of Gu and Novak, the different behaviour of malaria reduction with the increase of the buffer radius can be explained in term of the different gonotrophic cycle lengths used in the two models and also because in the model presented here the aquatic habitats are no uniformly distributed across the simulation landscape and are concentrated in the floodable areas in southeastern side of the village very close to the houses.

Possible future development of this study will be the design and the inclusion into the model of more policy-oriented scenarios in order to create a simulation tool that could be integrated into a malaria control scenario planning system for low incidence malaria areas. Such a simulation tool could be used to explore the effect of several local scale strategies to control or eliminate the malaria transmission like intra-household prevention, environment management prevention and effects of anti-malaria treatments.

## Abbreviations

ABM: agent-based model
DS: duration of Sporogony
LSM: larval source management

## Footnotes

1 Image of the Nanay River high level in the area around Padre Cocha: date of the image: 24 of March 2013 from Google Earth, 3°41′56.48″S, 73°16′43.04″W Image of the Nanay River low level in the area around Padre Cocha: date of the image: 12 of September 2013 from Google Earth, 3°41′56.48″S, 73°16′43.04″W.
